# Examining the role of anticipated enjoyment and intention in predicting attendance in exercise classes

**DOI:** 10.1186/s40359-025-03216-8

**Published:** 2025-08-05

**Authors:** Katharina Feil, Julian Fritsch, Susanne Weyland, Darko Jekauc

**Affiliations:** 1 Institute of Sports and Sports Science, Karlsruhe Institute of Technology, Karlsruhe, Germany; 2 Department of Sport Sciences, Goethe-University Frankfurt, Frankfurt, Germany

**Keywords:** Anticipation, Enjoyment, Intention, Exercise, Prospective

## Abstract

**Background:**

A large body of research shows that positive affect is related to higher physical activity levels. Building on this relation, a more novel approach suggests that also the anticipation of affective responses or more specific anticipated emotions may play a role for physical activity participation. Focusing on a specific emotion, the purpose of this study was to examine the relation between anticipated enjoyment, intention and exercise class attendance in a prospective design with three measurement occasions.

**Methods:**

In total, 363 adults (*M*_*age*_ = 32.28, *SD* = 14.11) were recruited from weekly exercise classes in Germany. Questionnaires for anticipated enjoyment and intention in relation to the next exercise class were sent to the participants in between two weekly exercise classes and the attendance of the next exercise class was subsequently assessed. Moreover, the maintenance of exercise classes was examined by assessing the attendance of five exercise classes after recruitment.

**Results:**

Logistic regression analyses showed that anticipated enjoyment was related to exercise class attendance, but this effect was non-significant when intention was added as an additional predictor. While intention was a significant mediator of the relation between anticipated enjoyment and exercise class attendance, anticipated enjoyment was not a significant moderator of the relation between intention and exercise class attendance. Linear regression analyses yielded that anticipated enjoyment was significantly related to exercise class maintenance, but this effect was non-significant again when intention was added as an additional predictor. The results suggest that anticipated enjoyment is not directly related to exercise participation but associated with intention which is a predictor of exercising.

**Conclusions:**

The results suggest that in line with previous theoretical models, anticipated enjoyment is related to intention which, in turn, is a predictor of exercising. Future research should include control variables to examine the impact of anticipated enjoyment on intention and physical activity behavior.

## Background

Physical inactivity has been associated with physical and psychological diseases such as obesity [1], cardiovascular diseases [2], metabolic syndrome [3] or dementia [4]. Therefore, the World Health Organization recommends regular moderate to vigorous physical activity of about 150 min per week for adults [5]. Despite the preventive health benefits of physical activity, many people fail to engage in regular physical activity. To achieve the recommended guidelines weekly exercise classes are particularly attractive for people who like to exercise in a group and with a professional trainer. However, in a study assessing the attendance of exercise classes over 13 weeks, it was shown that after the fourth week, only about half of the participants were still present [6]. This was the case despite constantly high intention rates regarding the next weeks exercise class participation suggesting that having the intention to participate does not secure regular attendance. Although intention is a relevant and necessary antecedent of physical activity, affective determinants of physical activity have received increasing attention in recent years. More specifically, the anticipation of affective responses was relevant for numerous health behaviors such as vaccination, cancer screening and physical activity in previous studies [7, 8].

### Theoretical considerations

Recent theories in exercise research suggest that affective variables influence physical activity behavior. The Affective Reflective Theory (ART) is central to this understanding, suggesting that two distinct processes—type-1 and type-2—govern physical activity behavior [9]. Type-1 processes are automatic, reflexive affective responses, such as acute affective reactions during exercise. In contrast, type-2 processes are slower, reflective, and require self-regulation, such as the intention to engage in physical activity. Affective variables can manifest as either type-1 or type-2 processes [9–11]. Type-1 processes occur without evaluative thinking, while type-2 processes involve cognitive appraisal, such as the anticipation of future affective responses to physical activity [10].

Anticipated affective responses are defined as affective responses that are expected to arise in the future in relation to an event [11]. In this definition, affective responses can be understood as an umbrella term for affective states, emotions, and moods. Affective states, which are commonly differentiated according to their valence and activation level [12], are often viewed as a facet of subjective well-being [13]. In contrast, emotions are typically viewed as specific psychophysiological reactions that are context-dependent and interwoven with cognitions [14]. In the present study, we focus on anticipated enjoyment as a specific emotion. According to the emotion-as-feedback theory by Baumeister et al. [14], the anticipation of emotions is central for decision making. The authors suggest that emotions give feedback about the world and these experiences build the basis for anticipating the future. Because this anticipation process is constantly running in our brain, Baumeister et al. [14] emphasize that the anticipation of emotions may predict future behavior even better than the actual previous emotional experiences.

Closely related to anticipated affect is affective attitude [15, 16] defined as the affective evaluation of physical activity [10, 11]. To assess affective attitude, participants are typically asked to indicate how enjoyable, boring or pleasant exercising would be for them [15, 16]. Affective attitude represents a general affective evaluation developed through repeated exercise experiences. In the present study, the focus on anticipated enjoyment differs from affective attitude in two ways. First, anticipated enjoyment was related to a specific exercise behavior (i.e., exercise classes) and second, the period of weekly exercise classes had just started suggesting that anticipated enjoyment was related to a new behavior for which an attitude needs to be built over time.

Based on the previously mentioned qualitative study, a theoretical model was proposed to explain how anticipated emotions may be related to physical activity behavior [17]. The authors suggest that individuals create specific expectations about future physical activity sessions based on previous physical activity experiences. These specific expectations (e.g., I will meet my best friend in the gym) can develop to anticipated emotions (e.g., I will enjoy the gym session) through appraisals (e.g., I am sure my best friend will show up or I like meeting him/her). These specific anticipated emotions may influence the intention (e.g., willingness to do the workout) as a proximal determinant of the behavior. Having the intention to exercise is one of the most prominent determinants of behavior in social-cognitive models such as the “Theory of Planned Behavior” (TPB, 18). In this theory, intention is proposed as the central predictor of behavior influenced by perceived behavioral control, subjective norm, and attitude. Whether anticipated affective responses may explain additional variance in intentions is still not fully understood [8].

### Anticipated affective responses and physical activity

Several reviews support the positive relation between reflective affect variables (e.g., affective attitude, anticipated affect) and physical activity behavior in different settings and ages [10, 19–23]. Regarding the influence of anticipated affective responses on physical activity, a recent scoping review suggests that especially positive anticipated affective responses such as anticipated enjoyment are positively related to physical activity behavior [8]. For example, in a prospective study, a positive correlation between positive anticipated affective responses during exercise and days of moderate physical activity four weeks later was found [24]. In the same study, positive anticipated affective responses after exercise were positively correlated with days of vigorous physical activity. While this study asked participants about their anticipated affective responses during and after exercise [24], another study asked participants about their anticipated affective responses if they successfully completed 90 days of physical activity [25]. They found that the anticipation of positive affective responses before the 90 days started was associated with a higher likelihood of physical activity adoption and maintenance [25]. Despite these rather consistent findings regarding the positive relation between positive anticipated affective responses and physical activity, in the present study, we focus on one specific anticipated emotion (enjoyment) related to exercise class engagement. For that, a validated questionnaire for physical activity enjoyment by only adapting the tense in the items was used [26]. In addition, tackling the question whether anticipated enjoyment is an independent predictor of physical activity behavior [8], the present study focuses on the complex interaction between anticipated enjoyment, intention and exercising including multiple measurement occasions of the behavior.

### Anticipated affective responses and intention

While a convincing amount of research focused on the relationship between anticipated self-conscious emotions (e.g., regret) and physical activity behavior, only some studies examined the relation between positive anticipated affective responses and intention. Two studies found a small [27] to moderate [24] correlation between positive anticipated affective responses and physical activity intention. Another study found that anticipated affect about an exercise prescription for the following week was significantly associated with the odds of “very strong intention” to follow through with the prescribed exercise program [28]. In a longitudinal study, anticipated enjoyment prior to a 30-minute workout only marginally predicted exercise intention after the workout [29]. These findings are consistent with the theoretical model proposed in the discussion of a qualitative study on anticipated emotions [17], in which intention was proposed as a mediator between anticipated affect and physical activity behavior. In addition, research focusing on other behaviors such as binge-drinking or eating-behavior suggest that anticipated emotions are related to reflected processes such as intentions or self-control which then influence behavior [30, 31].

The role of intention in physical activity behavior has been debated over the past decade. Traditionally, intention has been considered a central predictor of behavior. This was underpinned by a meta-analysis which has shown a correlation of approximately *r* =.48 between intention and physical activity [32]. Notably, observational studies show that a large change in intention results in a small effect on behavior [33, 34]. Therefore, many studies have focused on identifying moderators for the relation between intention and the subsequent behavior. With regards to the intention-physical activity relation, a recent systematic review found that anticipated regret (for missing physical activity opportunities) was a significant moderator of the intention-physical activity relation in four studies while only one study showed null findings [35]. Based on these findings, the authors proposed that thoughts about expected feelings can be considered as helpful moderators of the intention-physical activity relation compared to more instrumental judgements. However, Feil et al. pointed out in a scoping review on anticipated affect, it is not clear whether this moderation effect of anticipated regret is also evident for a positive emotion such as anticipated enjoyment [8]. That anticipated enjoyment may be a moderator of the intention-physical activity relation is underpinned by a large body of research showing that positive emotions such as enjoyment are positively associated with physical activity behavior [20, 21].

### The present study

**Fig. 1:**
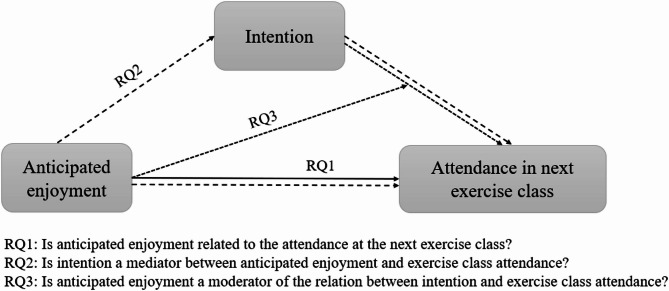
Research questions one to three. Note: RQ = research question

**Fig. 2:**
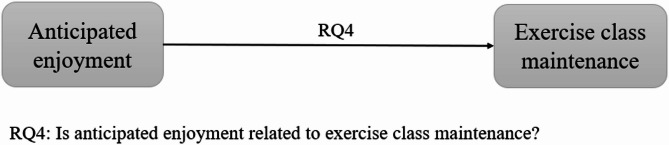
Research question four. Note: RQ = research question

While previous studies often focused on physical activity behavior in general, the present study assessed the attendance of weekly exercise classes as a specific exercise behavior. As Feil et al. [17] pointed out in their qualitative study, different anticipated emotions may be related differently to different physical activity behaviors. For example, one person may anticipate enjoyment when going for a run but anticipates disappointment when attending an exercise class, while for another person it is the other way around. Weekly exercise classes are characterized as organized classes with professional instructors in stable contexts and participants must actively register for an exercise class of their choice. These classes may differ in terms of intensity, content, complexity, instructors and environments, but they reflect real-life practice, as participants have different preferences. By focusing on a variety of exercise classes, we narrowed the scope of physical activity down to a specific, regular type, while still maintaining enough breadth to apply our findings to weekly exercise classes in general. Because we wanted to analyze the relation between anticipated enjoyment, intention and physical activity in different timeframes, we assessed exercise class attendance in a shorter and longer timeframe: one in which exercise class attendance was measured one to four days after anticipated enjoyment and intention, and another in which the exercise class attendance was assessed for the following four classes. The purpose of this study was to examine the relation between anticipated enjoyment, intention and exercising. For that four research questions have been outlined: (1) Is anticipated enjoyment related to the attendance at the next exercise class? (2) Is intention a mediator between anticipated enjoyment and exercise class attendance? (3) Is anticipated enjoyment a moderator of the relation between intention and exercise class attendance? (4) Is anticipated enjoyment related to exercise class maintenance? Based on the theoretical ideas and preliminary evidence presented above, we hypothesized that (1) anticipated enjoyment would be positively related to exercise class attendance, (2) intention would be a mediator between anticipated enjoyment and exercise class attendance, (3) anticipated enjoyment would act as a moderator of the relation between intention and exercise class attendance, and (4) anticipated enjoyment would be positively related to exercise class maintenance. Research questions one to three are shown in Fig. 1 with exercise class attendance as the dependent variable of the three research questions. Research question four is shown separately in Fig. 2 with exercise class attendance as the dependent variable.

## Materials and methods

The four research questions of the study were registered in Open Science Framework (OSF) prior to data collection on 24th April 2023. The university’s data security commissioner and ethics committee approved the study.

### Participants

Participants were recruited from weekly exercise classes at a German university and two sports clubs located in Germany. The university offers exercise classes to students and employees who can purchase a membership for exercise classes lasting for one semester (i.e., six months). Exercise classes in the sports clubs were organized in cycles with each cycle lasting ten to 12 weeks. Data was collected from April to December 2023. Exercise classes comprised a variety of fitness classes (e.g., Yoga, Pilates, Zumba, Full-body workouts) and sports in a non-competitive setting (e.g., Badminton, Volleyball, Basketball). Participants of these classes were approached and informed about the purpose of the study at the end of an exercise class. The day of recruitment took place between the second and fourth week of the exercise programs. Eligible participants had to be at least 18 years old, understand German, and sign the written consent. The total sample size of the study was 476 at the day of recruitment, from which 363 participants filled out questionnaires about their anticipated enjoyment and intention to participate in the next exercise class at the next measurement occasion and were thus eligible for the analyses of this paper. The mean age of these 363 was *M* = 32.28 (*SD* = 14.11), 61.4% were female, and 59.0% were students (2 missings). The majority of participants attended exercise classes at the university (69.2%) and most participants were recruited from yoga classes (17.3%), handball (14.9%), and full-body fitness workouts (12.7%).

### Procedures

**Fig. 3:**
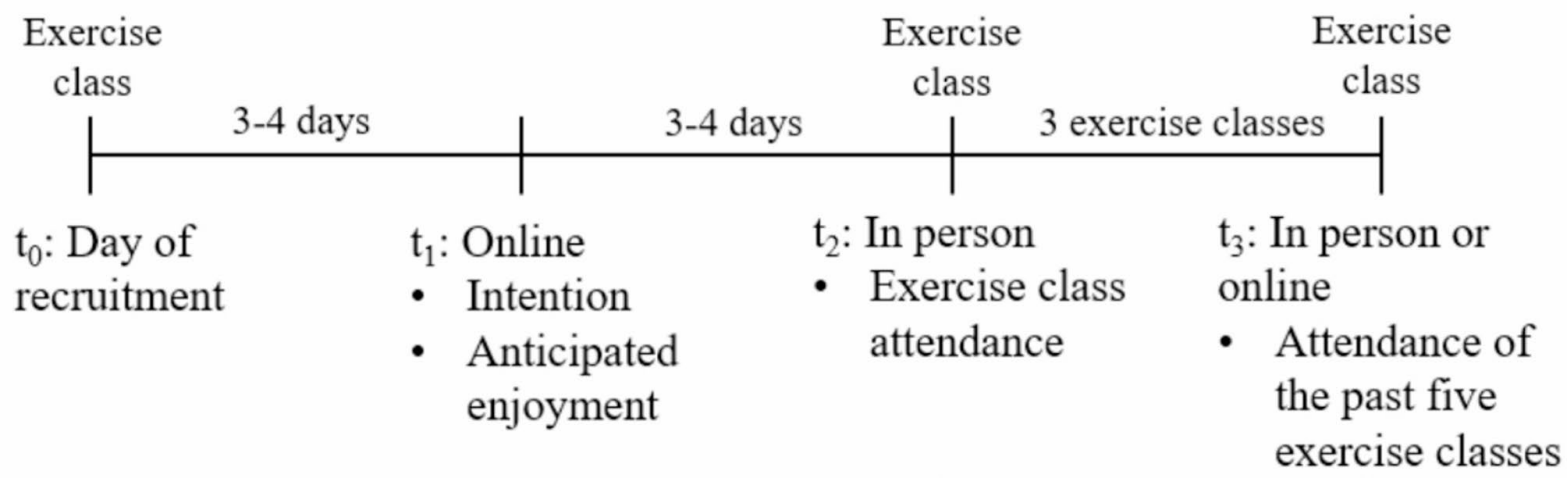
Study design and measurement occasions

Managers of the exercise programs and trainers of exercise classes were informed about the procedure of data collection. Participants were recruited directly after the exercise class and stated personal contact information which were stored separately from the collected data. Personal information and collected data could only be connected through individual codes. The first measurement occasion on the relevant variables (t1) took place three to four days after recruitment (t0) via e-Mail with a link to the online-platform SoSci-Survey measuring anticipated enjoyment and intention (see Fig. 3). We have chosen the gap of three to four days to avoid a rebound effect from the exercise class at t0 that might have happened if the participants were asked directly after the exercise class [36–38].

 Moreover, we decided to give the participants time to complete the questionnaire at t_1_ until 12am before the day of the next exercise class, because we wanted to avoid that t_1_ and t_2_ would coincide as a prospective study design was intended. The second measurement occasion (t_2_) took place in-person one week after the day of recruitment assessing exercise class attendance. The third measurement occasion (t_3_) took place after the fifth exercise class since the day of recruitment. This assessment was in-person after the exercise class or via SoSci-Survey in case participants did not attend the class at this specific day.^1^ Additionally, experienced enjoyment (with PACES-S, 26) and personality traits (with NEO-FFI, 39) were measured for other study purposes that are not part of this paper. Participants that answered questions at all measurement occasions were part of a lottery if they gave their consent for it. By recruiting from different exercise classes, we ensured that participants took part in exercise classes based on their own interests, since it was not known which classes would be included in the study and lottery of prizes at the beginning of the cycle. Prizes promoted the programs of the university and the sports clubs (e.g., free memberships for one semester or one cycle).

### Measures

As outlined in the procedure, several variables were assessed at different measurement occasions. The order of the questionnaires was randomized at the measurement occasion t_1_, because merely the assessment of these constructs at the same measurement occasion could have an effect on participants’ answers (e.g., 40, 41).

#### Anticipated enjoyment

Anticipated enjoyment was assessed with an adapted form of PACES-S [26, 42], which is a short version of the Physical Activity Enjoyment Scale (PACES, 43). This short version focuses on the subjective experience of enjoyment related to physical activity. The item stem was “When I imagine to participate in this class again, then I expect that…” and the four items were “I will enjoy it”, “I will find it pleasurable”, “It will be very pleasant”, and “It will feel good”. Participants answered on a likert scale ranging from one (strongly disagree) to five (strongly agree). Cronbach’s α was 0.85 in the present study.

#### Intention

The intention to participate in the next exercise class was assessed with two items “I intend to participate in this class again next week” and “I am sure that I will participate in this class again next week”. A seven-point response scale from “definitely not” to “definitely yes” was used for answering. The items had been widely used to measure intention [44]. Cronbach’s α was 0.74 in the present study while it was 0.83 in a study by de Bruijn et al. [44].

#### Exercise class attendance

A research assistant was present at t_2_ and checked the attendance.

#### Exercise class maintenance

The participation of the following five exercise classes since t_0_ was assessed at the third measurement occasion using two measures. First, one item assessed attendance of the four exercise classes between t_0_ and t_3_ asking “In the past four exercise classes I attended this class…”. Five answers were used to rank the participation (“not once”, “one time”, “two times”, “three times”, “all four times”). Second, a research assistant was present at t_3_ and checked the attendance at this day. Combining both measures, the variable exercise class maintenance ranged from 0 (no exercise class was attended since t_0_) to 5 (all exercise classes were attended since t_0_).

### Statistical analyses

Two a priori power analyses with G Power 3.1.9.4 were done, one for logistic regression analyses (research questions 1–3) and one for linear regression analyses (research question 4) with *p* <.05 and power (1-*ß*) adjusted to 0.80. For both analyses, effect size describing the relation between anticipated enjoyment and physical activity were used from a study by Kwan [24] showing a positive correlation between anticipated positive affect during exercise and the number of days being physically active on a moderate level (*OR* = 1.94 for logistic regression, *r* =.17 for linear regression). The power analysis for logistic regression analyses (X distribution binomial) revealed that *N* = 339 participants would be needed to reach the power of 0.80. The power analysis for linear regression analyses (fixed model, *R²* increase) with one predictor showed that *N* = 266 participants would be needed. Based on the available data of the participants, presented samples in the results comprise *N* = 363 participants for logistic regression analyses and *N* = 267 participants for linear regression analyses. We were able to reach this sample size by recruiting from a variety of exercise classes instead of one exercise class.

Preliminary analyses were carried out screening the data for missing values concerning item-nonresponse resulting in one missing value for one participant of the anticipated enjoyment scale (item 2). The little’s MCAR test was not significant suggesting that missingness was not related to the data (*χ²* = 1.01, *df* = 3, *p* =.800). The expectation-maximization algorithm for data imputation was used to avoid the deletion of the participant [45, 46].

To test the outlined research questions, four different statistical tests were carried out with SPSS (IBM SPSS Statistics 26). Regarding the first research question (i.e., assessing whether anticipated enjoyment would be related to exercise class attendance), we applied logistic regression analysis to examine the influence of anticipated enjoyment at t_1_ (independent variable) on attendance of the exercise class at t_2_ (dependent variable).

Regarding the second research question (i.e., assessing whether intention would mediate the effect of anticipated enjoyment on exercise class attendance), we performed a logistic regression analysis with anticipated enjoyment and intention both at t_1_ as predictors and attendance at t_2_. The reduction of the effect of anticipated enjoyment in the regression with intention as an additional predictor would indicate the presence of mediation. Additionally, we conducted a mediation analysis with Hayes’ PROCESS macro [47, 48]. Model 4 was applied which comprised a dependent variable Y (attendance at t_2_), an independent variable X (anticipated enjoyment at t_1_), and a mediator variable M (intention at t_1_).

For the third research question (i.e., assessing whether anticipated enjoyment would moderate the effect of intention on exercise class attendance), a logistic regression analysis was carried out including anticipated enjoyment at t_1_, intention at t_1_, and the product of anticipated enjoyment and intention at t_1_ as predictors of attendance at t_2_.

For the fourth research question (i.e., assessing whether anticipated enjoyment would be related to exercise class maintenance), a linear regression analysis was computed with anticipated enjoyment at t_1_ as a predictor (independent variable) and the exercise class maintenance at t_3_ (dependent variable). Significance level was set at *p* <.05 and reported *p*-values were divided by two for research questions one and four because we formulated directed hypotheses. Further, Nagelkerkes *R²* was reported in logistic regression analyses and adjusted *R²* in linear regression analyses. Because exercise class attendance was the dependent variable in research questions one to three and the maintenance of exercise class maintenance was the dependent variable in research question four, we did not employ a single model for all research questions and used a stepwise design.

## Results

### Descriptive statistics

From the included 363 participants at t_1_, 230 participants (63.4%) attended the exercise class at t_2_. The descriptive statistics of all variables and the correlations between them can be found in Table 1.

**Table 1:** Correlations and descriptive statistics of study variables

	1	2	3	4
1. Anticipated enjoyment t_1_	-	-	-	-
2. Intention t_1_	0.29*	-	-	-
3. Attendance at t_2_	0.11*	0.31*	-	-
4. Exercise class maintenance	0.16*	0.27*	0.33*	-
*M*	4.39	6.47	0.63	3.67
*SD*	0.66	1.02	0.48	1.26

Note: *p <.05

### Effect of anticipated enjoyment on exercise class attendance (Hypothesis 1)

The logistic regression analysis showed a significant effect of anticipated enjoyment on exercise class attendance (*OR* = 1.40, Nagelkerkes *R²* = 0.016, *χ²* = 4.1, *df* = 1, 95% *CI* = 1.011–1.927, *p* =.022). If the anticipated enjoyment scale increased by one, the odds for attending the next exercise class increased by 39.6%. Thus, Hypothesis 1 was supported.

### Intention as a mediator between anticipated enjoyment and exercise class attendance (Hypothesis 2)

When including intention as an additional predictor in the logistic regression, anticipated enjoyment was no more a significant predictor of exercise class attendance (*OR* = 1.06, *χ²* = 0.11, *df* = 1, *p* =.738). However, intention was a significant predictor of exercise class attendance (*OR* = 2.05, *χ²* = 24.20, *df* = 1, *p* <.001). This means that if the intention strength increased by one, the odds of attending the next exercise class doubled. Since the effect of anticipated enjoyment on exercise class attendance was not significant anymore when intention was added as a predictor, a mediation effect can be inferred.

To underpin the results, we conducted a mediation analysis using Hayes’ PROCESS macro yielding similar results. In the first model (Table 2) with intention as the dependent variable and anticipated enjoyment as the independent variable, anticipated enjoyment was a significant predictor (*B* = 0.441, *SE* = 0.078, *t*(1) = 5.652, *p* <.001). In the second model (Table 3) with exercise class attendance as the dependent variable and anticipated enjoyment and intention as independent variables, intention was a significant predictor (*OR* = 2.05, *SE* = 0.146, *p* <.001), while anticipated enjoyment was not a significant predictor anymore (*OR* = 1.06, *SE* = 0.187, *p* =.738).

**Table 2:** Effect of anticipated enjoyment on intention

Outcome variable: Intention at t_1_	Regression coefficient B	SE	t	*p*	Lower CI	Upper CI
Anticipated enjoyment t_1_	0.441	0.078	5.652	< 0.001	0.288	0.594
Intercept	4.537	0.346	13.130	< 0.001	3.858	5.217

Model summary: *R²* = 0.081, *R* =.285, *F* = 31.947, *df1* = 1, *df2* = 361, *p* <.001

**Table 3:** Effects of anticipated enjoyment and intention on exercise class attendance

Outcome variable: Attendance at t_2_	Regression coefficient B	SE	z	*p*	Lower CI	Upper CI	OR
Anticipated enjoyment t_1_	0.063	0.188	0.3348	0.738	− 0.303	0.428	1.06
Intention t_1_	0.719	0.146	4.9195	< 0.001	− 0.428	0.433	2.05
Intercept	-4.377	1.088	-4.0239	< 0.001	-6.509	-2.245	-1.66

Model summary: Nagelkerkes *R²* = 0.131, *-2LL* = 440.584, *df* = 2, *p* <.001

**Fig. 4:**
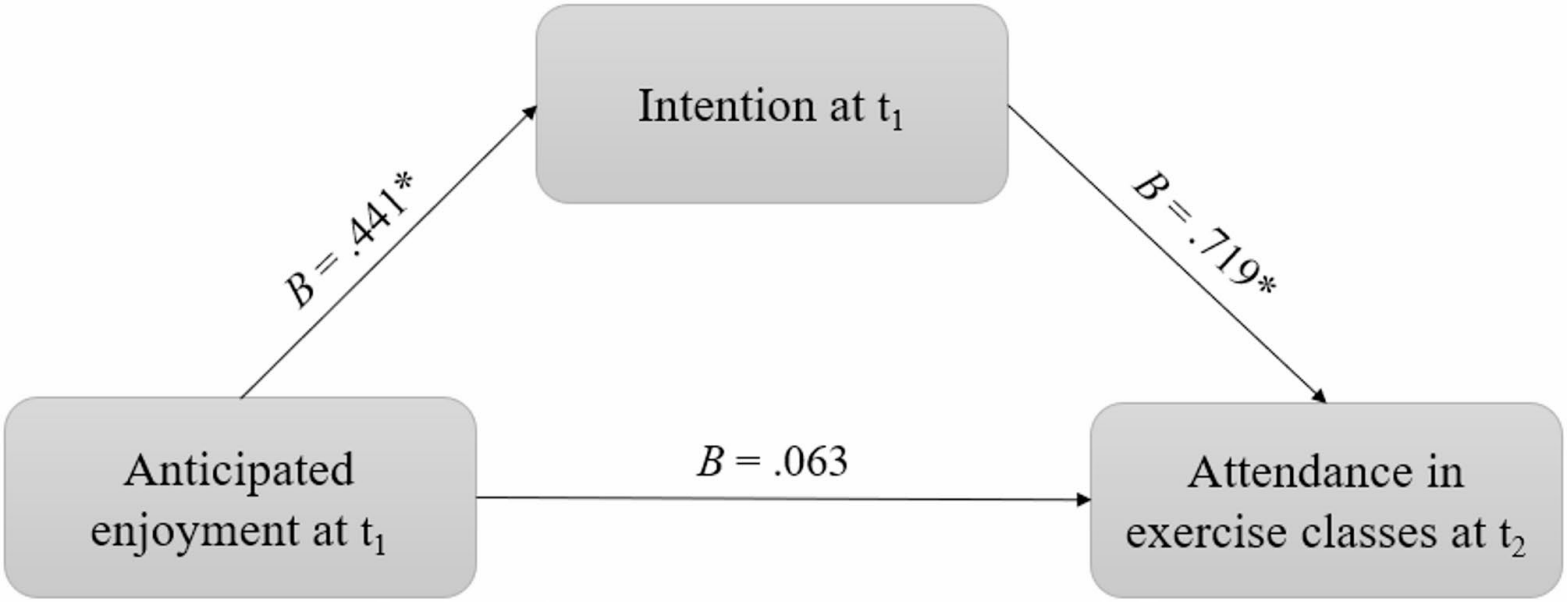
Results of mediation analysis. Note: *p <.05

The direct effect anticipated enjoyment at t_1_ on attendance at t_2_ was 0.063 (*SE* = 0.188, *z* = 0.3, *p* =.738, *CI* = − 0.303-0.428). The indirect effect of anticipated enjoyment via intention on exercise class attendance yielded a confidence interval from 0.114 to 0.137 confirming the mediation (*SE* = 0.114, *z* = 2.7, *p* =.006). Therefore, Hypothesis 2 was supported (see Fig. 4).

### Anticipated enjoyment as a moderator of the relationship between intention and exercise class attendance (Hypothesis 3)

The logistic regression analysis indicated that both predictors anticipated enjoyment (*OR* = 0.68, 95% *CI* = 0.091–5.070, *p* =.706) and intention (*OR* = 1.55, 95% *CI* = 0.449–5.387, *p* =.487), as well as their interaction (*OR* = 1.07, 95% *CI* = 0.790–1.453, *p* =.657) did not significantly influence the odd of attending the next exercise class (Table 4). Therefore, Hypothesis 3 was not supported.

**Table 4:** Results of moderation analysis

Outcome variable: Attendance at t_2_	Regression coefficient B	SE	χ²	df	*p*	OR
Anticipated enjoyment	− 0.388	1.026	0.143	1	0.706	0.68
Intention	0.441	0.634	0.484	1	0.487	1.55
Interaction term	0.069	0.155	0.197	1	0.657	1.07
Intercept	-2.572	4.124	0.389	1	0.533	0.08

### Effect of anticipated enjoyment on exercise class maintenance (Hypothesis 4)

The linear regression analysis revealed a small but significant effect of anticipated enjoyment on attendance of the next five exercises classes since t_0_ (*β* = 0.157, *b* = 0.301, *t*(1) = 2.580, adjusted *R²* = 0.021, 95% *CI* = 0.071 − 0.530, *p* =.005). If anticipated enjoyment increased by one unit, the number of exercise class attendances increased by 0.301. Anticipated enjoyment explained 2.1% of variance in the sum of exercise class attendances. Thus, Hypothesis 4 was supported. Additionally, we conducted an exploratory analysis with intention as an additional predictor in the linear regression yielding the same result as in the logistic regression analysis. While intention was a significant predictor of the attendance of the next five exercise classes (*β* = 0.245, *b* = 0.322, *t*(2) = 3.832, adjusted *R²* = 0.069, 95% *CI* = 0.161 − 0.502, *p* <.001), the effect of anticipated enjoyment was not apparent anymore (*β* = 0.065, *b* = 0.125, *t*(2) = 1.017, 95% *CI* = 0.117 − 0.366, *p* =.310).

## Discussion

The purpose of this study was to examine the relation between anticipated enjoyment, intention, and exercising. Anticipated enjoyment was related to exercise class attendance, however, when intention was added as an additional predictor this effect was not significant anymore. Subsequent analyses suggest that anticipated enjoyment may be a predictor of intention, which in turn influences exercise class attendance. The moderation analysis, in which anticipated enjoyment was postulated as a moderator of the relation between intention and exercise class attendance, did not yield significant results. Moreover, anticipated enjoyment was significantly related to the exercise class maintenance. However, exploratory analyses revealed that the effect was no longer significant when intention was included as an additional predictor in the regression.

The relatively low correlation and significant relation between anticipated enjoyment and exercise class attendance is in line with previous research [24, 25]. However, these studies did not consider possible moderators or mediators of this relationship and the differences in the study design should be considered when comparing results. First, the reference point of the anticipated affective construct may differ between studies as suggested in a recent scoping review [8]. In the present study, anticipated enjoyment was related to how participants would feel during the next exercise class (very similar in 24), while in a previous study positive anticipated affect was related to how participants would expect to feel after completing a 90-day physical activity period [25]. Second, the timeframe was different as in the present study anticipated enjoyment was related to an event occurring within the next day(s). On the contrary, in the previously mentioned study, anticipated affect was related to an event occurring after 90 days [25].

The positive relation between anticipated enjoyment and exercise class attendance is also consistent with the theoretical considerations by a more general emotion theory by Baumeister et al. [14]. In the emotion-as-feedback theory, it is assumed that anticipated emotions are related to future behavior, which was tested in the present study in form of exercise class attendance. However, a theoretical model in exercise psychology by Feil et al. [17] suggested, anticipated emotions may be rather related to proximal determinants of exercise behavior such as intention than the behavior itself, which would explain why the effect of anticipated enjoyment was not significant anymore when intention was added as an additional predictor in the analysis [17]. The relation between intention and exercise class attendance superimposed the effect of anticipated enjoyment, suggesting that the attendance of exercise classes as a more planned behavior may have been predicted by more regulative processes like intention (as suggested in [49, 50]). Attendance was assessed in the first half of the exercise programs in week two to four, when automatic processes may not have been developed yet, supporting the notion that attendance in the present study may have been a more planned behavior. Furthermore, a study focusing on different health behaviors showed that individuals who base their intention on affect rather than on cognitions have a stronger intention-behavior relation [51]. This relation was mediated by the temporal stability of intention, suggesting that intention based on affect may be more stable over time and therefore more likely to be translated into behavior.

Consistent with previous research [24, 27, 29], anticipated enjoyment was positively associated with the intention to exercise. Furthermore, the mediating effect of intention suggests that anticipated enjoyment may be a predictor of intention. Ajzen [52] postulated that anticipated affect is closely related to affective attitudes and therefore does not add value to the prediction of intention (see also 53). However, meta-analyses have shown that anticipated affect and affective attitude are separate constructs, as they independently predicted intention and behavior [16, 54–56]. This was also the case when controlling for the variables subjective norms, attitude and perceived behavioral control [16] and especially in health-protective behaviors such as physical activity [56]. However, we need to emphasize that we did not include these control variables which is why we cannot conclude from the results that anticipated enjoyment is an independent antecedent of intention.

We also tested whether anticipated enjoyment was a moderator of the intention-behavior relation. In contrast to previous studies showing that anticipated regret was a significant moderator of the intention-behavior-relationship (e.g., 41, 57), we did not find such an effect in the present study. In a study that also examined exercise class attendance as an outcome variable over a period of 13 weeks, it was tested whether experienced affect after exercise classes was a moderator of the relation between intention and the re-participation in weekly exercise classes [58]. No significant effects were found either on the between- or on the within-person level.

Furthermore, our study found that anticipated enjoyment was associated with exercise class maintenance. Based on the results of the second research question, we exploratively added intention as a predictor and found that the effect of anticipated enjoyment on exercise class maintenance was not significant anymore when intention was added. This finding supports again the theoretical idea that anticipated enjoyment predicts intention but not behavior itself [17]. According to a recent scoping review on anticipated affect [8], only one study has examined the relation between anticipated affective responses and a 90 day physical activity program [25]. It was shown that positive anticipated affective responses after the 90-day period predicted whether or not a participant would exercise regularly during that period. However, they did not include intention as an additional variable and the anticipated affective response was related to achievement of completing 90 days of regular exercising. Another study found that experienced affect predicted intentions at both the between-person and within-person levels [58]. Similar to our findings, experienced affect predicted re-attendance, but this effect was non-significant when intention was added as a predictor. Moreover, a study that analyzed the attendance of weekly boot-camp classes showed that automaticity was a significant predictor of class participation of four consecutive weeks, while intention was not [59]. The participants attended the boot-camp for four weeks prior to the study suggesting that more automatic processes may become more relevant for class attendance over time (as proposed in 60). In the present study, automatic processes were not included but could have influenced exercise class maintenance.

### Strengths and limitations

The present study examined the role of anticipated affect as an understudied psychological construct in exercise class attendance and maintenance. A prospective study design allowed for the analysis of the relation between anticipated enjoyment and the next exercise class attendance and maintenance, which included a total of five exercise classes. The strength of this approach lies in its external validity as the exercise classes were part of real exercise programs provided by local institutions. However, this study comes with several limitations. First, we were unable to control for the participants’ physical activity level, exercise class characteristics [61] and instructor competencies [62] that may have influenced the relation between anticipated enjoyment, intention, and exercise class attendance. In the same vein, control variables that could have influenced the relation between anticipated enjoyment and exercise class attendance (e.g., momentary affect, remembered affect) or the relation between anticipated enjoyment and intention (e.g., perceived behavioral control, attitude, subjective norms) are missing. In addition, variables such as experienced affect, habit or past behavior could have also served as mediators between anticipated enjoyment and exercise class attendance but were not assessed in this study. Second, analyses were conducted on the between-person level, because the data was not suitable for within-person analysis. Accordingly, no statements can be made about the extent to which changes in anticipated enjoyment could have caused changes in intention, exercise class attendance and maintenance. Similarly, it would have been more appropriate to establish a temporal order between anticipated enjoyment, intention, and behavior to analyze the mediation effect, rather than measuring anticipated enjoyment and intention simultaneously. Moreover, the used method does not allow to consider the dynamics of anticipated enjoyment. These aspects should be considered in future studies. Third, participants received the online survey at t_1_ three to four days after t_0_ and had time to complete the questionnaire until 12 am before the day of the next exercise class (t_2_). Thus, the exact time point of t_1_ varied between participants. Fourth, participants pre-registered and paid for the entire exercise cycle, which may have led to self-selection and reduces the generalization of results. In addition, the incentive of winning a free cycle of exercise classes may have contributed to confounding the results as it may have attracted particularly physically active participants. Fifth, consistent with previous research [26, 58] participants reported high levels of anticipated enjoyment and intention, potentially leading to ceiling effects. In addition, the short form of PACES contains only positively worded items, which may have led to measurement bias.

As a consequence of these limitations, future studies may benefit from using ambulatory assessment methods that allow within-person variation based on the repeated measures of anticipated enjoyment, intention, and behavior [63]. Frequent assessments of anticipated enjoyment and intention leading up to the moment of executing the behavior would provide a better understanding of the interplay between anticipated enjoyment, intention and behavior. Moreover, future studies should include additional variables that may influence this interplay such as other affective determinants of exercising, previous exercise experience and other predictors of intention.

## Conclusion

The present study examined the role of anticipated enjoyment in exercise behavior and showed that anticipated enjoyment was indirectly related to exercise behavior via intention. This finding supports the relevance of intention as a predictor of behavior and suggests that anticipated enjoyment is a predictor of intention but not an independent predictor of the behavior itself. However, if anticipated enjoyment is an independent and relevant antecedent of intention is still unclear due to missing control variables. Future research is encouraged to include control variables that are relevant for intention and physical activity behavior. Additionally, collecting intensive longitudinal data would facilitate within-person analyses, potentially yielding more nuanced insights into these relationships.

## Data Availability

The datasets used and analysed during the current study are available from the corresponding author on reasonable request.

## Footnotes

1 In the preregistration of the study the third measurement occasion was planned five weeks after the day of recruitment. We changed it to five exercise classes since the day of recruitment because some exercise classes were cancelled and we wanted to ensure that every participant had the same number of exercise classes between the day of recruitment and the third measurement occasion.

## Declarations

### Abbreviations

We have not abbreviations in the text to declare.
